# Action Direction of Muscle Synergies in Three-Dimensional Force Space

**DOI:** 10.3389/fbioe.2015.00187

**Published:** 2015-11-13

**Authors:** Shota Hagio, Motoki Kouzaki

**Affiliations:** ^1^Japan Society for the Promotion of Science, Tokyo, Japan; ^2^Laboratory of Neurophysiology, Graduate School of Human and Environmental Studies, Kyoto University, Kyoto, Japan

**Keywords:** muscle activity, electromyogram, non-negative matrix factorization, force fluctuations, mechanical pulling direction, cross-correlation analysis

## Abstract

Redundancy in the musculoskeletal system was supposed to be simplified by muscle synergies, which modularly organize muscles. To clarify the underlying mechanisms of motor control using muscle synergies, it is important to examine the spatiotemporal contribution of muscle synergies in the task space. In this study, we quantified the mechanical contribution of muscle synergies as considering spatiotemporal correlation between the activation of muscle synergies and endpoint force fluctuations. Subjects performed isometric force generation in the three-dimensional force space. The muscle-weighting vectors of muscle synergies and their activation traces across different trials were extracted from electromyogram data using decomposing technique. We then estimated mechanical contribution of muscle synergies across each trial based on cross-correlation analysis. The contributing vectors were averaged for all trials, and the averaging was defined as action direction (AD) of muscle synergies. As a result, we extracted approximately five muscle synergies. The ADs of muscle synergies mainly depended on the anatomical functions of their weighting muscles. Furthermore, the AD of each muscle indicated the synchronous activation of muscles, which composed of the same muscle synergy. These results provide the spatiotemporal characteristics of muscle synergies as neural basis.

## Introduction

The fundamental problem in motor control is how the central nervous system (CNS) controls the immense number of variables in the musculoskeletal system (Bernstein, 1967). To simplify the redundancy, the CNS may modularly organize the muscles through the hard-wired neural circuit referred to as muscle synergy (Tresch et al., 1999; d’Avella et al., 2003; Ting and Macpherson, 2005; Hagio and Kouzaki, 2014). To clarify the underlying mechanisms in motor control based on muscle synergies, it is important to examine how muscle synergies are represented and modulated in the neural circuitry (motor level) and to investigate whether muscle synergies function as the actuator to produce movement (task level) (Alessandro et al., 2013). Many researchers statistically calculated task-dependent muscle synergies from electromyogram (EMG) dataset in motor level (d’Avella et al., 2006, 2008; Torres-Oviedo and Ting, 2007, 2010; Hug et al., 2010; Roh et al., 2012, 2013; Hagio et al., 2015), whereas model-based approaches showed the low dimensionality in the task level (Berniker et al., 2009; Neptune et al., 2009; Allen and Neptune, 2012). To uniformly identify the relationship of the low dimensionality between motor and task levels, it is necessary to quantify the net contribution of individual muscle synergies in the task space.

Several approaches were conducted to demonstrate correlations between muscle synergy recruitment levels and biomechanical outputs. During perturbed standing, functional muscle synergies were calculated, which reflect the mapping of the endpoint force vector (Torres-Oviedo et al., 2006; Chvatal et al., 2011). Previous research estimated the mechanical contribution of each muscle synergy (called as synergy-to-force mapping) by assuming the linear relationship between EMG (further linearly decomposed into muscle vectors of muscle synergies) and endpoint force in isometric condition (Berger and d’Avella, 2014). These techniques were advantageous to quantify the force vector produced by each muscle synergy in the force space. However, the force vectors did not contain the temporal contribution of muscle synergies, which is important property to regard muscle synergies as neural basis. Although our previous study directly compared the spatiotemporal correlation between the activation coefficients of muscle synergies and endpoint force fluctuations during voluntary isometric conditions, demonstrating the significant correlation between them (Hagio and Kouzaki, 2015), the mechanical contribution of muscle synergies in the task space was not estimated. An appropriate approach has been taken using *EMG-weighted averaging* (EWA) method (Kutch et al., 2010; Imagawa et al., 2013). This was formulated as a non-invasive technique instead of the spike-triggered averaging (STA), i.e., a well-established method to extract the force associated with single motor unit (SMU) contractions, based on the hypothesis that surface EMG is indeed analogous to a superposition of SMU action potentials and its cross-correlation with endpoint force should produce the equivalent of an average spike-triggered force averaged across multiple motor units (Kutch et al., 2010). In this study, we developed this technique to evaluate the action direction (AD) of muscle synergies, which represented the net contribution of individual muscle synergies in the three-dimensional endpoint force. It should be noted that we assumed the neural basis of muscle synergies: the estimated activation of muscle synergies represents the summation of the individual basis constructing muscle synergies, which might have been regarded as spinal interneuron in the previous studies (Hart and Giszter, 2010; Overduin et al., 2014).

In the muscle synergy hypothesis, the primary problem is still whether the CNS actually modulates muscle synergies in the neural circuit. Many empirical findings showed the neural basis of muscle synergies by examining the relationship between statistically calculated muscle synergies and activation of spinal interneuron in frogs (Hart and Giszter, 2010) or activation of motor cortical neurons in rhesus macaques (Overduin et al., 2014). However, it is reported that low dimensionality as statistically calculated muscle synergies might be due to task or biomechanical constraints (Kutch and Valero-Cuevas, 2012). Accordingly, the problem remains controversial (Bizzi and Cheung, 2013). In the concept of the synchronous muscle synergy, which is discriminated from other muscle synergy models, such as time-varying muscle synergy (d’Avella et al., 2003), muscles organized in the same muscle synergies may be synchronously activated. Therefore, cross-correlation analysis will lead to the correlation between the activation of the target muscle and endpoint force, which are generated by the muscles grouped in the same muscle synergy; the AD of a muscle will reflect the mechanical contribution not only of the muscle but also of the other muscles synchronously activated due to a muscle synergy as hard-wired modular controller. Hence, the examination of the relationship among the ADs of muscles weighted by the same extracted muscle synergy will make it possible to approach identifying the neuronal basis of muscle synergies. In this study, we examined the presence of muscle synergies by calculating AD of each muscle.

Consequently, the main purpose of the present study was to quantify the contribution of muscle synergies in the task space. To this end, we estimated the AD of muscle synergies during multi-directional force generation in three-dimensional force space. Furthermore, we verified the hypothesis of the neural basis of muscle synergies by examining the relationship between the ADs of individual muscles and the estimated structure of muscle synergies which the relevant muscles belong to. This study demonstrates the relationship of low dimensionality due to muscle synergies between in the motor and task levels.

## Materials and Methods

### Subjects

Five male subjects voluntarily participated in this study. Their mean (±SD) age, height, and body mass were 23.8 ± 1.1 years, 173.9 ± 3.8 cm, and 67.4 ± 6.5 kg, respectively. All subjects were healthy, had no history of any neurological disorder, and had corrected-to-normal vision. Subjects provided written informed consent to participate in the study after receiving a detailed explanation of the purposes, potential benefits, and risks associated with participation. All procedures used in this study were in accordance with the Declaration of Helsinki and approved by the Committee for Human Experimentation at the Graduate School of Human and Environmental Studies, Kyoto University.

### Experimental Setup

**Figure 1:**
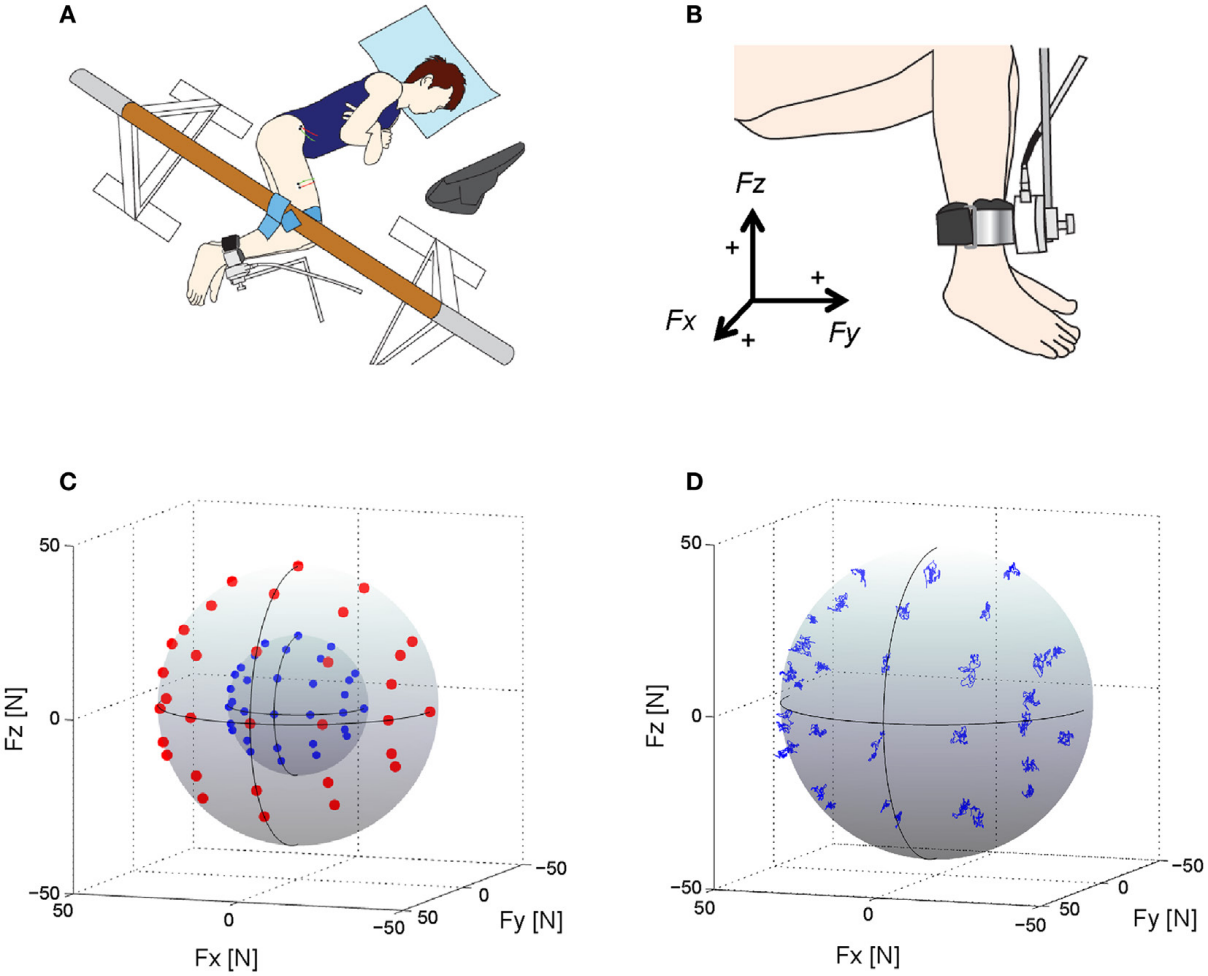
**Experimental setup, target directions and force trajectories**. **(A)** An overhead view of the experimental setup. Subjects lay on their left side on a bed with the right leg supported horizontally by a sling. Visual feedback of produced and target forces was displayed to the subject on a computer screen (Hagio and Kouzaki, 2014). **(B)** Using a tri-axial force transducer attached to the subject’s right ankle, three-dimensional forces, *F*_x_, *F*_y_, and *F*_z_, were measured (Hagio and Kouzaki, 2014). The positive values of three axes are corresponding to hip abduction (+*F*_x_), knee extension (+*F*_y_), and hip flexion (+ *F*_z_) movement directions, respectively. **(C)** Thirty-two desired target force directions (blue dots: 20 N, red dots: 40 N) in the three-dimensional force space. **(D)** The force trajectories across each target direction in the force intensity of 40 N for a representative subject.

Each subject laid on their left side on a bed with the right leg supported horizontally by a sling (Figure 1A; Hagio and Kouzaki, 2014, 2015). The knee and hip joints were applied with the angles of 90° from full extension. Isometric endpoint forces surrounding the right ankle were produced for a total of 10 s at 2 different intensities (20 and 40 N) in each of 32 different directions in the three-dimensional force space (Figure 1C); in total, 64 trials were randomly conducted with a rest period of 30 s between each trial and of 10 min between 2 blocks which is composed of 32 trials, respectively. The directions were equally distributed in 30° increments along horizontal plane to cover the anterior side on this plane. On sagittal plane, force was applied from six directions (0°, 30°, 60°, 90°, 120°, and 135°) considering the knee extension torque and/or hip joint torque (Hof, 2001). We then measured isometric endpoint forces, which were composed of three force vectors, *F*_x_, *F*_y_, and *F*_z_ referring to hip abduction–adduction, knee extension–flexion, and hip flexion–extension movements, respectively (Figures 1B,D), using a tri-axial force transducer (LSM-B-500NSA1, Kyowa, Tokyo, Japan) attached to the subject’s right ankle (Kouzaki et al., 2002; Hagio et al., 2012). The resultant force vector was calculated based on the three force vectors, i.e., *F* = *F*_x_ + *F*_y_ + *F*_z_; the resultant vector length represented the intensity of the force, i.e., $|F|\text{=}\sqrt{F_{\text{x}}^{2}+F_{\text{y}}^{2}+F_{\text{z}}^{2}}.$ In each trial, the subjects viewed the produced force vector and the desired force vector as a target on a visual display.

### Electromyography

Surface EMGs were recorded from eight muscles mainly activated in the task space in this study: the rectus femoris (RF), vastus lateralis (VL), vastus medialis obliquus (VMO), vastus medialis longus (VML), vastus intermedius (VI), sartorius (SR), adductor longus (AL), and gluteus medius (GM) (Hagio and Kouzaki, 2014, 2015). EMGs were recorded using bipolar Ag–AgCl electrodes. Each electrode had a diameter of 5 mm, and the inter-electrode distance was 10 mm. We used a small inter-electrode distance to prevent cross-talk between neighboring muscles (Imagawa et al., 2013). A reference electrode was placed on the lateral epicondyle of femur. The EMG signals were amplified (MEG-6116M, Nihon-Kohden, Tokyo, Japan) and band-pass filtered between 5 and 1000 Hz. All electrical signals were stored with a sampling frequency of 2000 Hz on the hard disk of a personal computer using a 16-bit analog-to-digital converter (PowerLab/16SP; AD Instruments, Sydney, NSW, Australia). The raw EMG traces were high-pass filtered at 35 Hz using a zero-phase-lag fourth-order Butterworth filter, after which they were demeaned, rectified, and low-pass filtered at 40 Hz (Chvatal et al., 2011). The filtered traces were then divided into 100 time bins per second and averaged across each bin (i.e., resampled at 100 Hz). The same procedures were conducted across each corresponding rest period, and the difference between the two traces served as the net EMG (Hagio and Kouzaki, 2015).

For the extraction of muscle synergies, the muscle activity data for each muscle were assembled to form an EMG data matrix. We first constructed the EMG data matrix (*M*), which consisted of temporal sequence for 10 s of each muscle activity in each trial, i.e., 8 muscles × 64,000 variables (32 directions × 2 force levels × 10 s × 100 samples). The EMG values of each muscle were normalized to the maximum value for all of the muscles across all desired directions such that each value was between 0 and 1. Then, each muscle data vector was normalized to have unit variance to ensure the activity in all muscles was equally weighted.

### Extraction of Muscle Synergies

We extracted muscle synergies from the data matrix of the EMG recordings (*M*) using non-negative matrix factorization (NMF) (Lee and Seung, 1999; Tresch et al., 1999; Hagio and Kouzaki, 2014, 2015; Hagio et al., 2015) as following equation:

$$M\text{=}\sum_{\text{i}=1}^{\text{N}}W_{\text{i}}C_{\text{i}}+\varepsilon \text{ (}W_{\text{i}}\geq 0,C_{\text{i}}\geq 0\text{)}$$

where *W*_i_ represents the contribution of each muscle to synergy i, and an individual muscle may contribute to multiple synergies. The composition of the muscle synergies does not change among the conditions, but each synergy is multiplied by a scalar activation coefficient (*C*_i_) that changes among conditions: the column of *C*_i_ consisted of 64,000 variables (32 directions × 2 force levels × 10 s × 100 samples). ɛ is the reconstructed error. The synergy weighting and activation coefficient matrices were normalized such that the individual muscle-weighting vector was the unit vector.

To select the smallest number of muscle synergies (*N*_syn_) that resulted in an adequate reconstruction of the muscle responses, we extracted between 1 and 8 muscle-weighting matrices of muscle synergies and activation coefficient matrices from the EMG data matrices that were obtained from each subject. We subsequently verified the goodness-of-fit between the original (*M*) and reconstructed $\left(M_{\text{r}}=\sum_{\text{i}=1}^{\text{N}}W_{\text{i}}C_{\text{i}}\right)$ data matrices as the amount of total variability explained (*R*^2^) depending on the number of muscle synergies (*N*). We used a linear regression procedure (d’Avella et al., 2006) to determine *N* value after which the *R*^2^ curve is approximately straight as assuming that the increase of *R*^2^ with adding *N* value is due to noise-based variation. We performed linear regression on the entire *R*^2^ curve and progressively removed the smallest *N* value from the regression interval. We then compared the mean square residual errors of the different regression lines and selected the least *N* value (*N*_syn_), a mean squared error in the regression line from which to the maximum *N* value was <10^−4^. For *N*_syn_ muscle synergies, both muscle-weighting and activation-coefficient matrices were defined.

For the verification that the extracted muscle synergies depend on not the methodological but physiological factors, it is needed to judge whether the resultant dimensionality in the muscle activation space using the NMF analysis was lower than the chance level or not. To this end, EMG data matrix was constructed using the shuffled EMG data across each muscle. It should be noted that these shuffled EMG data contained the same value, range, and variance for each muscle although the relationships between muscle activations were removed. We then calculated *R*^2^ value between the original and reconstructed EMG data matrices across each of one to eight muscle synergies.

### Grouping of Similar Muscle Synergies Across Subjects

Functional sorting of the muscle synergies across each subject was initially performed by grouping muscle synergies based on the values of cosine similarity (*r* > 0.78; *p* < 0.01) to that of an arbitrary reference subject using an iterative process (Hagio and Kouzaki, 2014, 2015). If two synergies in one subject were assigned to the same synergy group, we defined a pair of synergies with the highest correlation as the same group of synergies. Subsequently, an averaged set of similar muscle synergies for all subjects were computed, and the similarity between the averaged muscle synergies and each synergy grouped across the subjects was quantified.

### Evaluating Action Direction of Muscle Synergies and Muscles

**Figure 2:**
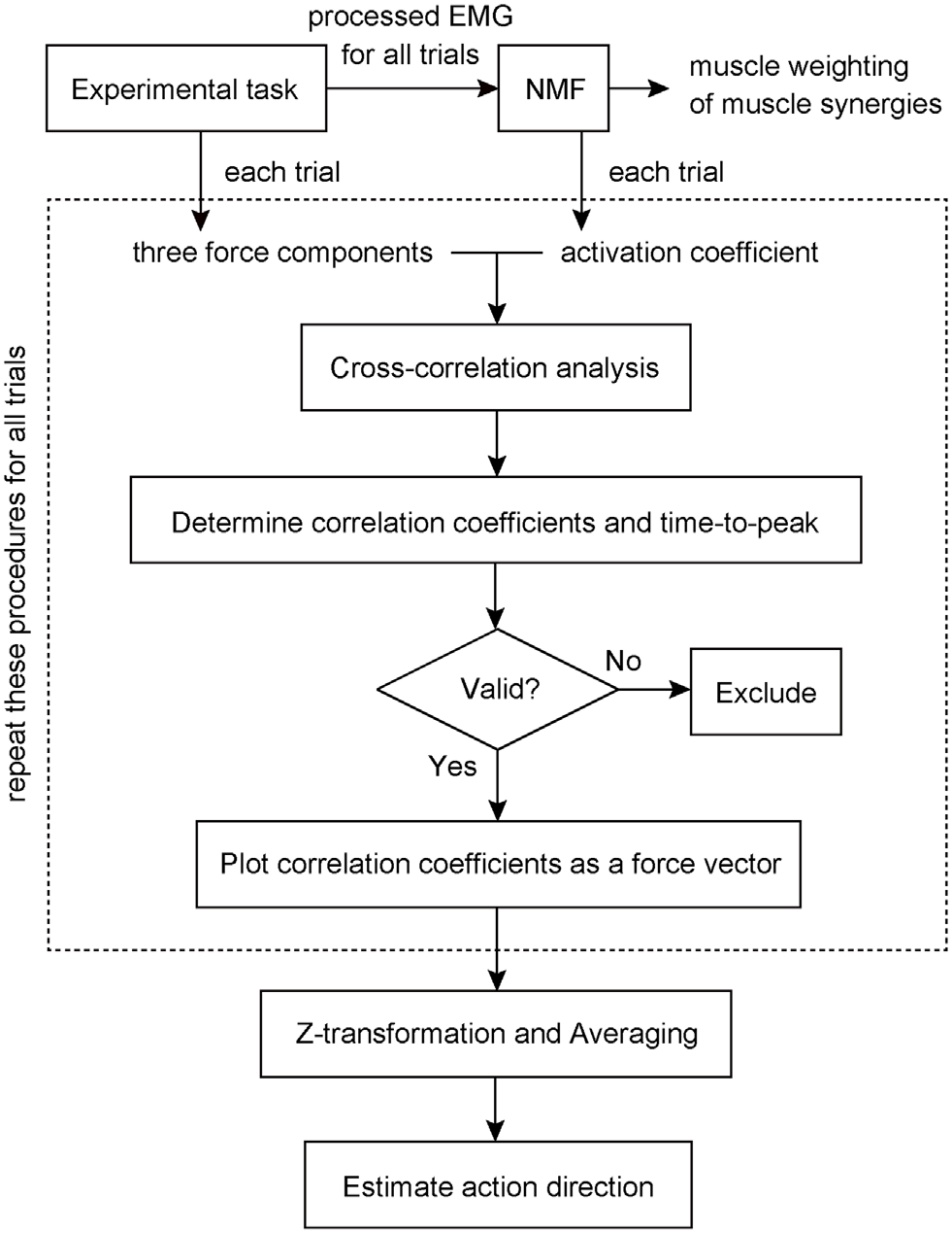
**Block diagram to estimate action direction of a muscle synergy**. Muscle weightings and activation coefficients of muscle synergies were first extracted from processed electromyogram (EMG) data for all trials using non-negative matrix factorization (NMF). The cross-correlation analysis was then performed between the traces of three force components and activation coefficient of a muscle synergy recorded and estimated in each trial to determine each correlation coefficient and the time-to-peak. If the correlation was physiologically valid, the correlation vector was plotted in three-dimensional space as a force vector. These procedures (surrounded by a dashed line) were repeated for all trials. Finally, we estimated the action direction of the muscle synergy, which was the averaging of force vectors after the correlation coefficients underlying the force vector were transformed with Fisher’s *Z* transformation. The details were described in the Section “Materials and Methods.”

**Figure 3:**
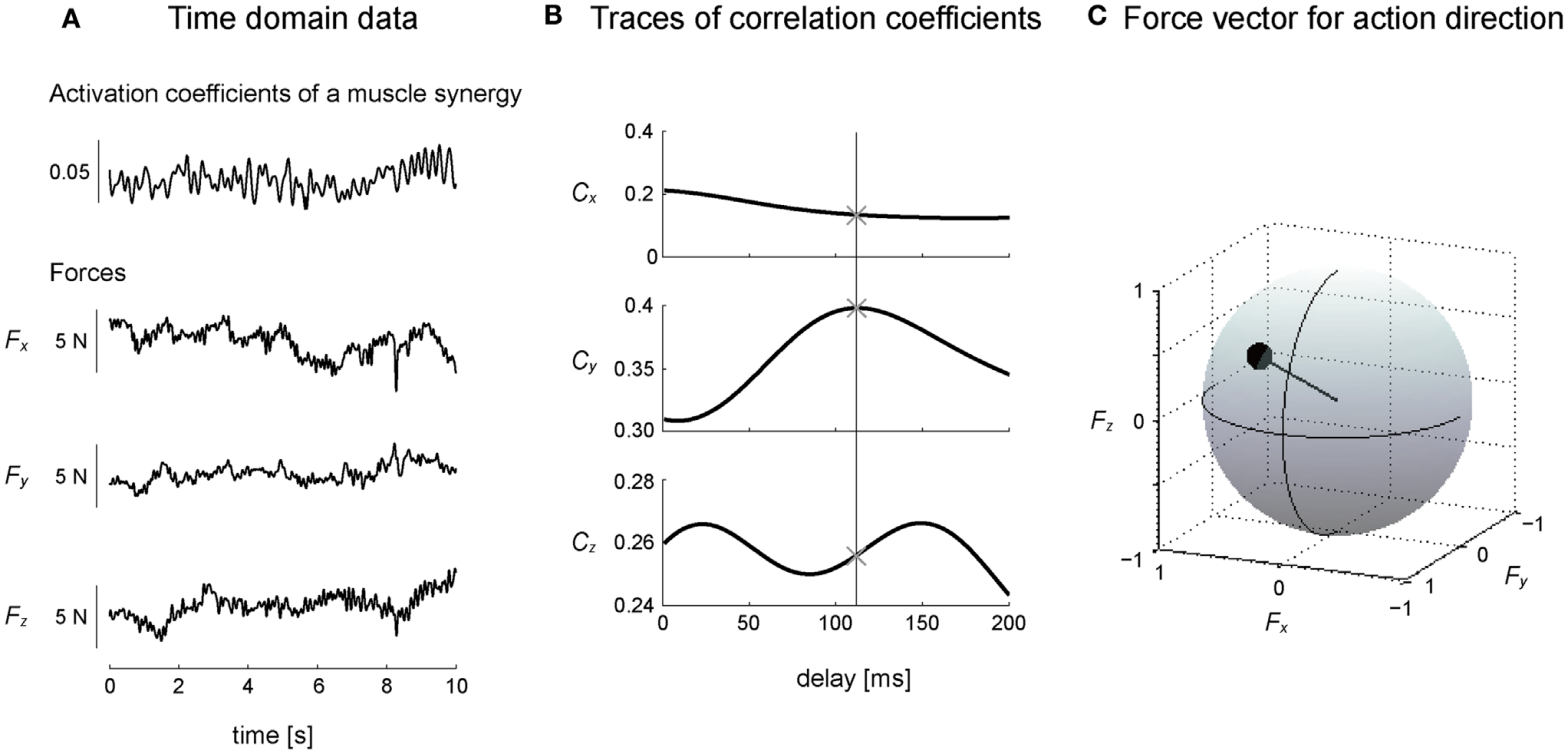
**Calculation of action direction**. **(A)** A representative trial is shown in the time domain, where the subject maintained a desired isometric force around the right ankle, which arose from the activation of a muscle synergy. **(B)** Cross-correlation between activation coefficients of a muscle synergy and each force component yielded the traces of correlation coefficients peaking at time lag (between EMGs constructing activation coefficients of the muscle synergy and force) about 100 ms. The time-to-peak is shown as a vertical line (see detail in Section “Materials and Methods”). ×, the point in which the most peak magnitude of the correlation coefficients was observed. **(C)** Force vector was determined as a unit vector based on the correlation coefficients of three components in three-dimensional force space. The correlation coefficient was described as the radius of a plot on the surface of unit sphere. These procedures were corresponding to a block diagram surrounded by a dashed line in Figure 2.

We estimated the three-dimensional AD of muscle synergies and muscles by developing EWA method (Kutch et al., 2010; Imagawa et al., 2013). Figure 2 provides a diagram of how the method operates. The EWA is based on a cross-correlation of EMGs and force signals. Such analysis was performed over an approximately steady period of force fluctuations lasting 10 s out of the time course used in prior analysis. We used a series of estimated activation coefficients of each muscle synergy (*C*_i_; i = 1, 2, … , *N*) along with three force components (*F*_x_, *F*_y_, and *F*_z_) for cross-correlation analysis (Figure 3A). For the estimation of ADs of muscles, cross-correlation analysis was performed between the processed surface EMGs from individual muscles and each of the corresponding three force components. Each correlation coefficient was first quantified temporally and spatially based on a time lag from 0 to 200 ms, during which the traces reached its peak magnitude (Figure 3B). We used the time lag, on which the most peak magnitude of the three was estimated, to define the time-to-peak and used the corresponding time lag to define the net correlation coefficient of remaining components. According to the correlation coefficients of each component, the force vector in the three-dimensional space was determined across each trial (Figure 3C). Then, we defined the AD of the muscle synergy or muscle, which was the averaging of force vectors for all selected trials (see detail below) after the correlation coefficients underlying the force vector were transformed with Fisher’s *Z* transformation (Fisher, 1934).

To verify the physiological validity of the ADs, we considered the electromechanical delay (EMD) of each muscle. The EMD was referred to as the time lag between EMG and mechanical force response (Cavanagh and Komi, 1979; Norman and Komi, 1979), corresponding to the time-to-peak of cross-correlation in this study. Furthermore, to increase a validity of this technique, we adopted the trials, which were comprised in three time bins around the peak time bin of the histograms across each muscle, for estimating force vectors (see Results). If the same peak time bins were observed in histograms, we selected the time bin, which was close to 100 ms. In the case of muscle synergies, we determined the correlation coefficients of the time-to-peak value between 50 and 150 ms, which indeed reflected the EMD of muscles constructing the muscle synergies, based on the EMD of lower limb muscles in the previous study (Vos et al., 1990) and in this study (see Result).

### Methodological Identification of Action Direction

**Figure 4:**
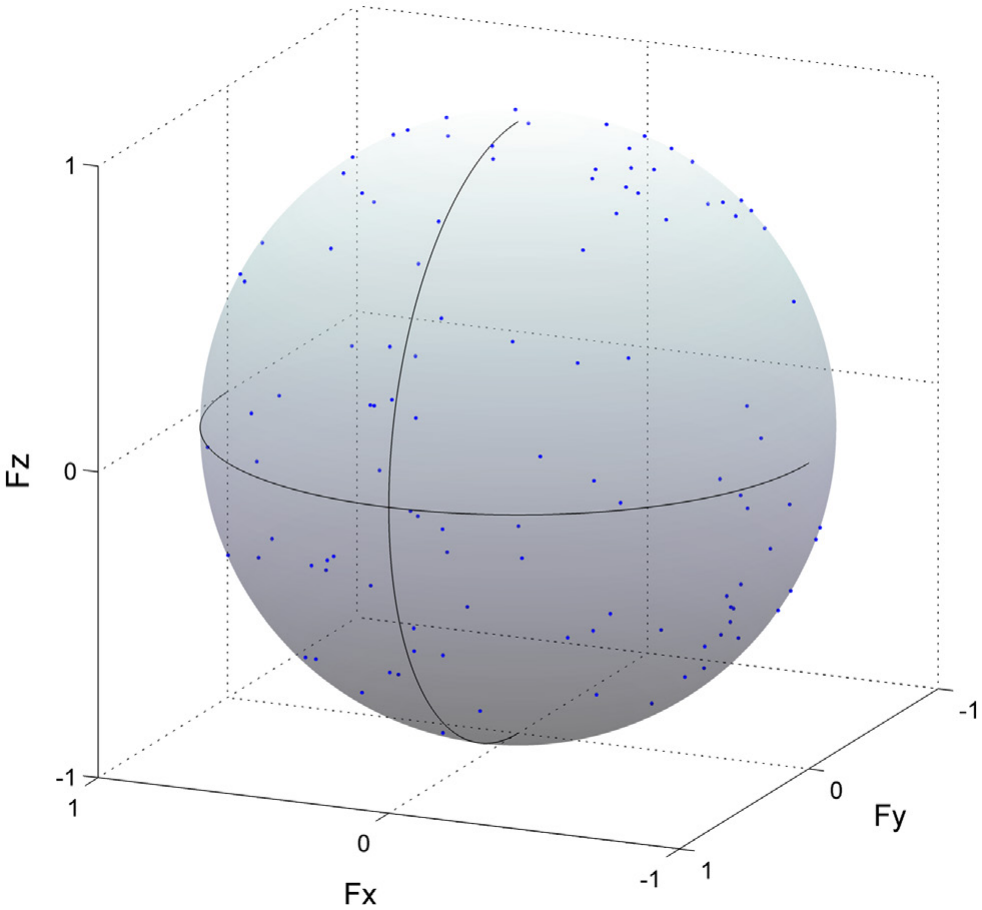
**Methodological identification of action direction**. Force vectors (blue dots) estimated from cross-correlation analysis between the shuffled electromyogram (EMG) traces and force responses for all trials in the rectus femoris are shown (detail in Section “Materials and Methods”). This procedure was repeated 100 times using bootstrapping to resample each shuffled EMG data. The length of each vector was normalized with their unit vectors, and the vectors distributed on the surface of unit sphere. The correlation coefficient was represented as the radius of each plot. The positive values of three axes are corresponding to hip abduction (*F*_x_), knee extension (*F*_y_), and hip flexion (*F*_z_) movement directions, respectively.

To validate the analysis for the estimation of ADs, we performed methodological identifications. We verified that the distribution of force vectors as a result of correlation coefficients was not due to a secondary product of the methodology but due to a physiological factor, i.e., the relationship between the muscle activation and endpoint force. To this end, we calculated force vectors with the same procedure as estimating ADs of muscle synergies (or muscles), using the three force components and shuffled activation traces of muscle synergies (or EMG data) in which temporal sequences were shuffled across each muscle synergy (or muscle) (Figure 4). The force vectors, which time-to-peak value was physiologically meaningful, i.e., between 50 and 150 ms based on the previously calculated EMD (Vos et al., 1990), were adopted. We then quantified the distribution of the force vectors as a resultant vector length [$R=||\bar{r}||;$ norm of the force vector averaged for each force vector (*r*), i.e., length of AD vector (Fisher, 1995)]. This procedure was repeated 100 times using bootstrapping to resample each shuffled activation trace of muscle synergy (EMG data) (Efron, 1993). We then estimated 95% bootstrap confidence intervals for the overall resultant vector length. If a resultant vector length calculated by actual dataset was out of the 95% confidence interval, the distribution of the force vectors was not determined by chance but included physiological information.

## Results

### Directional Tuning of EMG Activity

**Figure 5:**
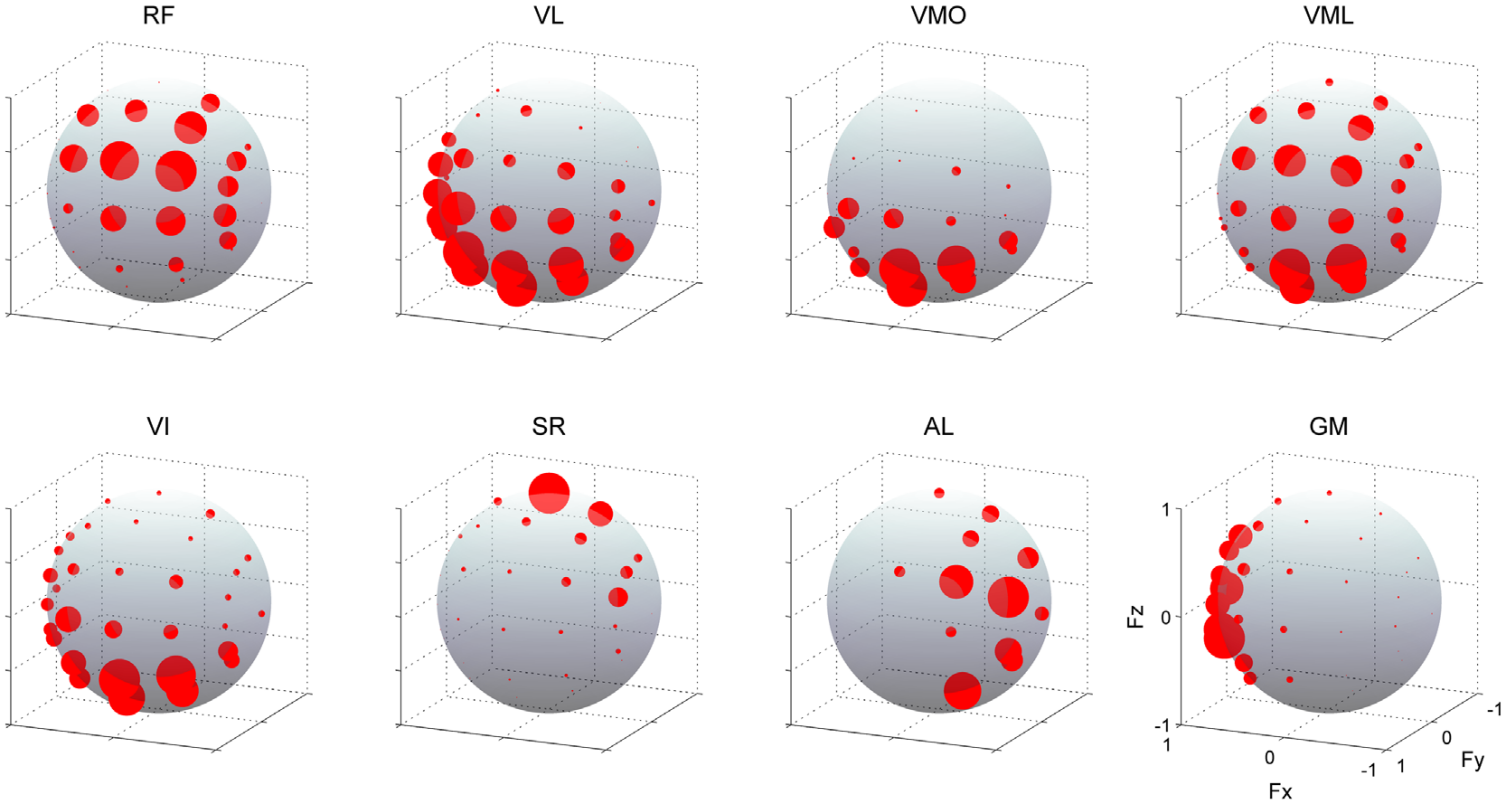
**Muscle activities**. EMG activities across each muscle. The amplitudes of EMGs were represented as the radius of each plot distributed on the surface of unit sphere. Data are shown across each target direction in the force intensity of 40 N for a representative subject. Muscle names are indicated in an abbreviated form: RF, rectus femoris; VL, vastus lateralis; VMO, vastus medialis obliquus; VML, vastus medialis longus; VI, vastus intermedius; SR, sartorius; AL, adductor longus; GM, gluteus medius. The positive values of three axes are corresponding to hip abduction (*F*_x_), knee extension (*F*_y_), and hip flexion (*F*_z_) movement directions, respectively.

Figure 5 shows the muscle activations across each target direction in a representative subject. The activation of each muscle was broadly and specifically tuned with three-dimensional force direction. RF and VML were predominantly activated for between forward (+*F*_y_) and upward (+*F*_z_) force directions, which required knee extension and hip flexion torques, whereas VL, VMO, and VI were mainly activated forward (+*F*_y_) and close to downward (−*F*_z_) directions. It should be noted that the net knee extension torque, which does not involve the hip flexion or extension torques, was biased toward this direction on the force space. Hence, these mono-articular knee extensors produce the net knee extension torque. In the case of SR, AL, and GM, they generated hip flexion, adduction, and abduction torques, respectively.

### Muscle Synergy

**Figure 6:**
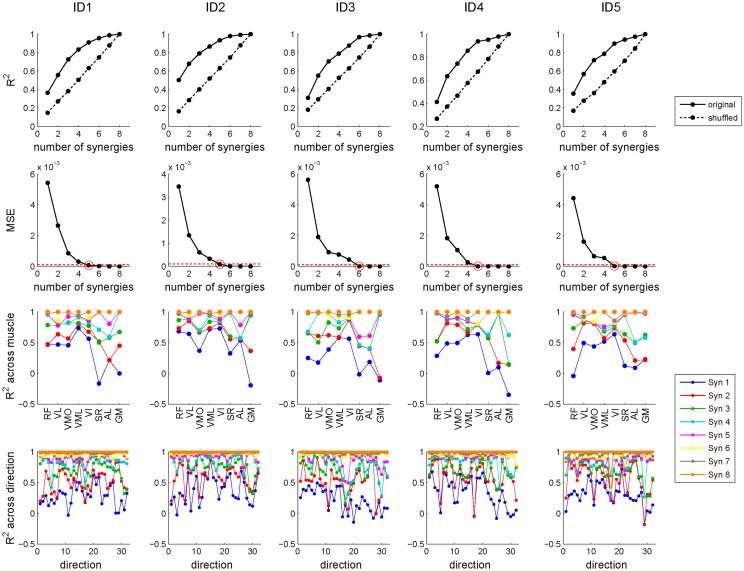
***R*^2^ value for determining the number of muscle synergies**. Top: the amount of total variability explained (*R*^2^) as a function of the number of synergies obtained from original (solid line) and shuffled (dotted line) EMG datasets across each subject. Second: mean square residual error (MSE) of the regression line on *R*^2^ curve from that number of muscle synergy to the maximum was computed. We selected the least number of muscle synergies (red circle), which MSE was <10^−4^ (red dash line). Third: *R*^2^ value across each muscle. Each line represents the *R*^2^ value of the certain number of muscle synergies. Bottom: *R*^2^ value across each of 32 target directions. Each line represents the *R*^2^ value of the certain number of muscle synergies. Syn, synergy.

**Figure 7:**
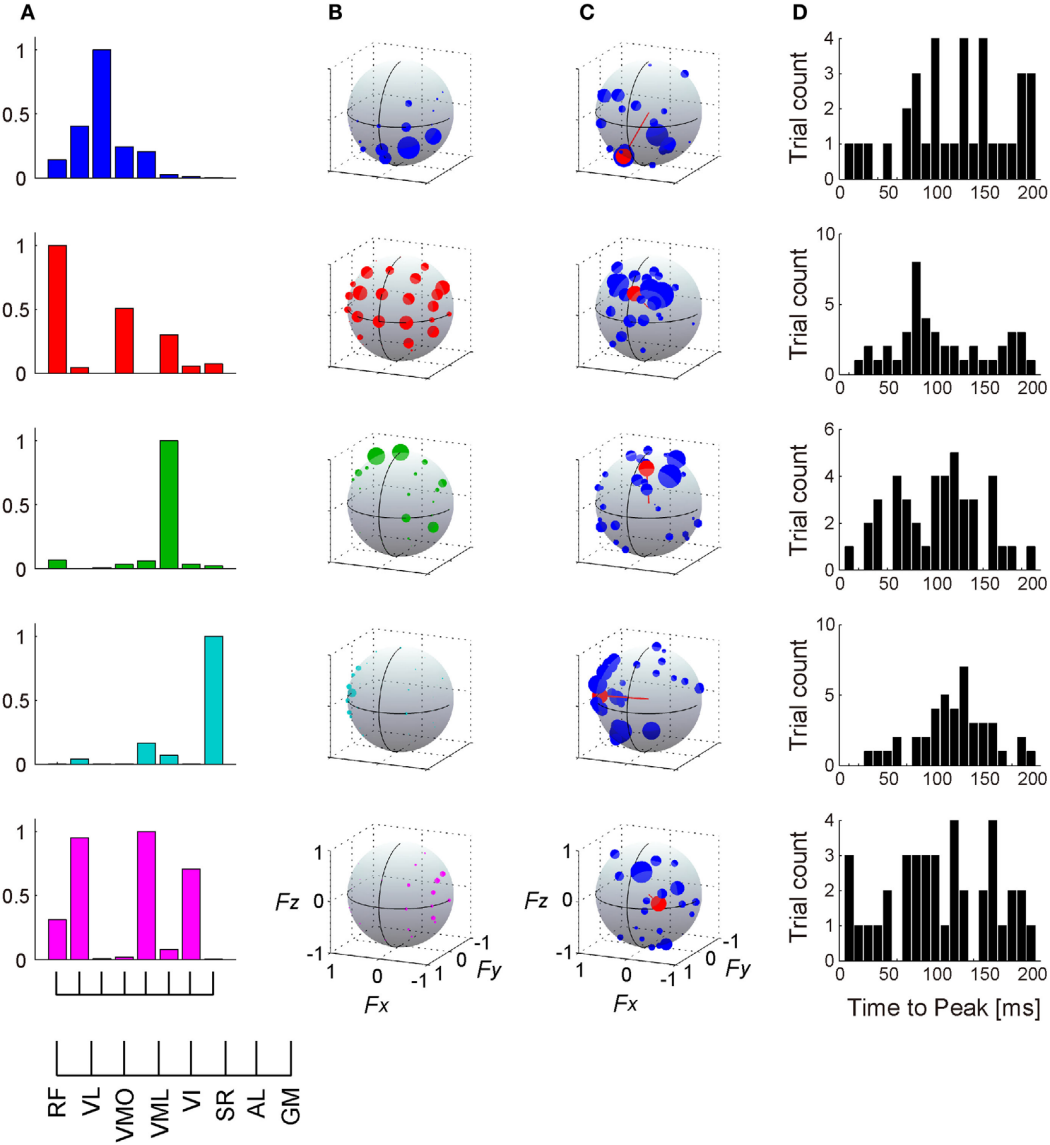
**Muscle synergies and their action directions**. The muscle weightings **(A)** and activation coefficients across each target direction in the force intensity of 40 N **(B)** of 5 extracted muscle synergies are shown in a representative subject. **(C)** The force vectors (blue) and action direction (AD; red) across each muscle synergy. The length of each vector was uniformed with their unit vectors, and the correlation coefficient was described as the radius of each plot distributed on the surface of unit sphere. The positive values of three axes are corresponding to hip abduction (*F*_x_), knee extension (*F*_y_), and hip flexion (*F*_z_) movement directions, respectively. Data shown are for selected trials (see detail in Section “Materials and Methods”). **(D)** Histograms of time-to-peak value obtained from cross-correlation analysis, representing the time lag between the onset of activation of muscle synergy and force responses (exactly, the delay between the onset of activation in muscle level and the force responses).

In this study, we extracted five or six muscle synergies which accounted for 92.9 ± 2.75% of the total data variability (*R*^2^) in the five subjects, and the *R*^2^ value for same number of synergies were definitely higher than the case of shuffled dataset across each muscle (Figure 6: top). Additionally, the data were sufficiently reconstructed across each muscle and each target direction, as determined by *R*^2^ averaged for all muscles and all directions: 92.3 ± 2.86 and 91.2 ± 4.24%, respectively (Figure 6: third and bottom). Figures 7A,B show five extracted muscle synergies and their activation coefficients across each target direction in a representative subject, respectively. The synergy *W*_1_, which was mainly constructed by mono-articular knee extensors (VL, VMO, and VI), was activated in forward (+*F*_y_) and downward (−*F*_z_) directions, i.e., the range around net knee extension direction, and around medial direction (−*F*_x_). The synergy W_2_, which contained RF, VML, and SR, was dominant for forward (+*F*_y_) and upward (+*F*_z_) directions generated by both knee extension and hip flexion torques and was also broadly activated in medial (−*F*_x_) and lateral (+*F*_x_) directions. The synergy *W*_3_ was mainly composed of SR, and activated around upward direction (+*F*_z_) produced by hip flexion torque. The synergy *W*_4_ having GM dominantly contributed to lateral force (+*F*_x_), which was generated by hip abduction torque. The synergy *W*_5_, which was constructed by RF, VL, VI, and AL, was activated around medial direction (−*F*_x_) produced by hip adduction torque.

### Action Direction of Muscle Synergy

Figure 7C represents the AD of each muscle synergy (red), which was defined as the averaging for individual force vectors (blue) resulting from cross-correlation analysis. We verified the significance in the distribution of the force vectors across each muscle synergy (*p* < 0.05; see detail in Section “Materials and Methods”). The ADs were approximately corresponding to the activation range of the muscle synergies: the synergy *W*_1_ contributed around net knee extension torque [(0.215, 0.680, −0.701)]; (*F*_x_, *F*_y_, *F*_z_)]; the synergy *W*_2_ was dominant for knee extension and hip flexion torques (−0.093, 0.850, 0.519); the synergy *W*_3_ was mainly activated for hip flexion torque (−0.180, 0.507, 0.843); the synergy *W*_4_ dominated hip abduction torque (0.999, 0.013, −0.048); and the synergy *W*_5_ generated knee extension, hip flexion, and hip adduction torques (−0.567, 0.818, 0.103). Figure 7D shows the time-to-peak histograms of each synergy at a time lag of 0 to 200 ms, which represents the time lag between the activation onset of muscle synergy and the onset of mechanical force response. Each muscle synergy had the peak time bin around a time lag of 100 ms [117.2, 103.3, 98.5, 115.2, and 101.4 (ms); mean value in *W*_1–5_, respectively).

**Figure 8:**
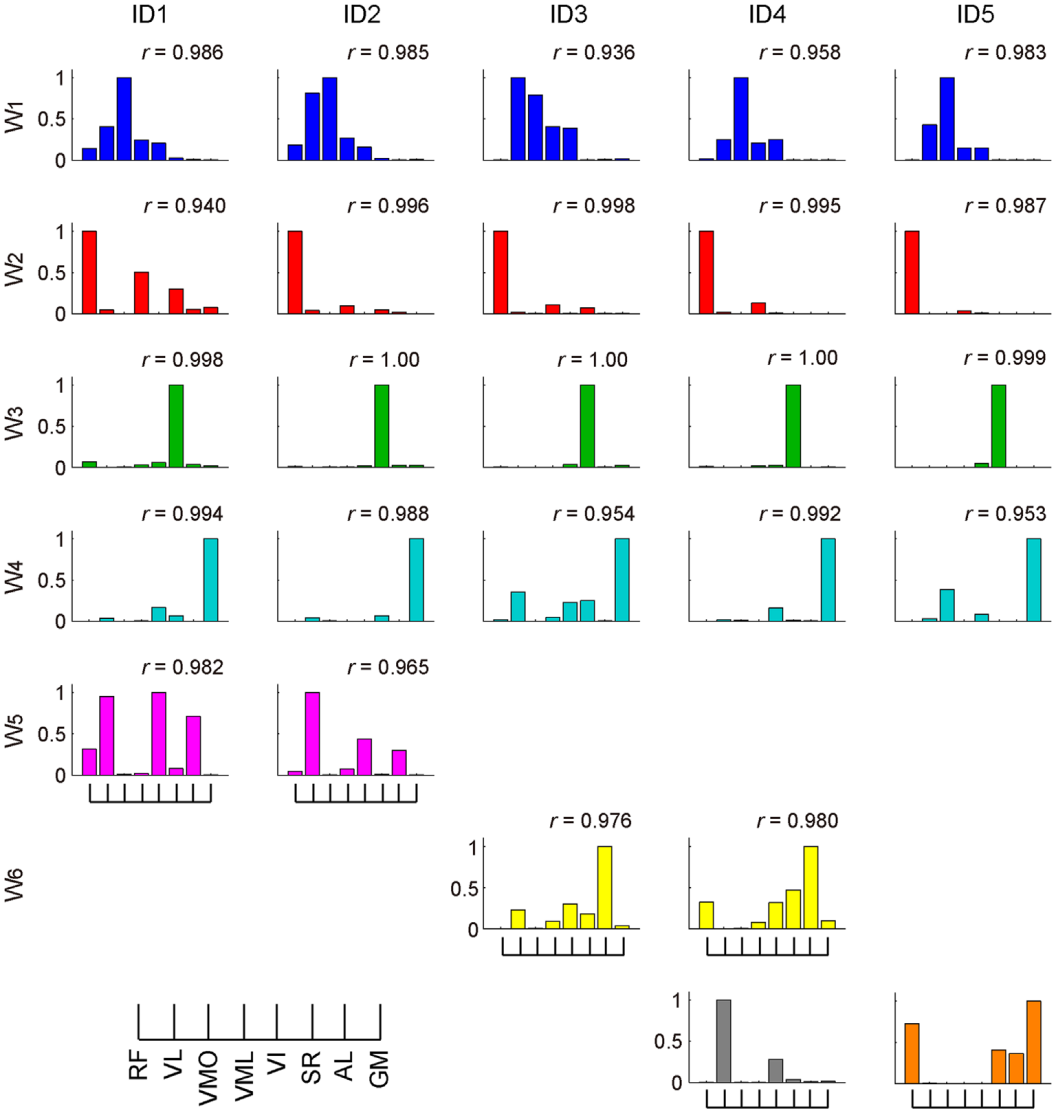
**Muscle synergies across each subject**. The muscle-weighting vectors of the muscle synergies across each subject are shown. The *r* value represents cosine similarities between the averaged muscle synergies estimated from the initial sorting and each original synergy grouped across each subject (see Materials and Methods). The synergies across each subject were grouped into six groups (*W*_1–6_) and two subject-specific muscle synergies (last row; gray and orange).

**Figure 9:**
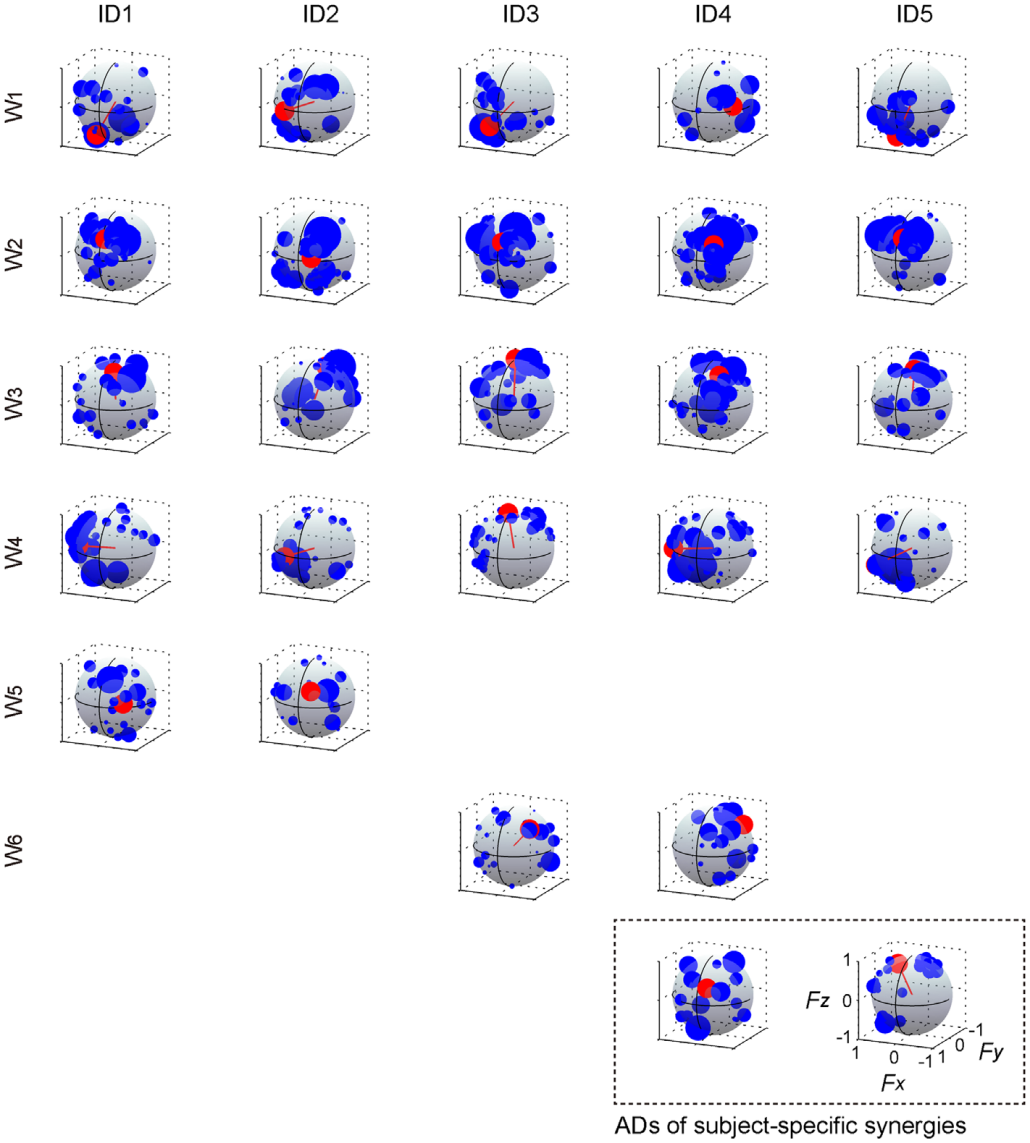
**Action direction of muscle synergies across each subject**. The ADs (red) and force vectors (blue) across each subject. The length of each vector was uniformed with their unit vectors, and the correlation coefficient was described as the radius of each plot distributed on the surface of unit sphere. The order of the panels was corresponding to those in Figure 8. The positive values of three axes are corresponding to hip abduction (*F*_x_), knee extension (*F*_y_), and hip flexion (*F*_z_) movement directions, respectively.

The muscle weighting and ADs of muscle synergies for all subjects are shown in Figures 8 and 9, respectively. The synergy *W*_1_, which was mainly constructed by mono-articular knee extensors, i.e., VL, VMO, VML, and VI, was extracted from all subjects with high similarity (*r* > 0.936). The ADs of the synergy *W*_1_ were distributed in the similar direction generated by knee extension torque in four of five subjects, whereas the AD of one subject (ID4) denoted more medial direction (−*F*_x_) than the others. The synergy *W*_2_ weighting RF and VML was observed in all subjects (*r* > 0.940). The AD of the synergy *W*_2_ was similar across each subject, which directional force was produced by the combination of hip flexion and knee extension torques. The synergy *W*_3_ dominantly composed of SR was included in all subjects (*r* > 0.998). The ADs of the synergy *W*_3_ were consistently directed approximately hip flexion direction (+*F*_z_) for all subjects. The synergy *W*_4_, which was constructed by the combination of GM and other muscles, was similar across all subjects (*r* > 0.953). The ADs of the synergy *W*_4_ mainly denoted the lateral direction (+*F*_x_), but in one subject (ID3) the AD was biased to the hip flexion direction (+*F*_z_) because of the influence of SR. The synergies *W*_5_ and *W*_6_, which commonly contained AL, were extracted 2 of 5 subjects, respectively (*r* > 0.965 and *r* > 0.976, respectively). The ADs of these synergies were distributed in the medial direction (−*F*_x_). The subject-specific synergies were observed in two subjects, the ADs of which depended on the composition of these muscle synergies. These results demonstrated the robustness and specificity of muscle synergies and their ADs across each subject.

### Action Direction of Muscles on the 3-D Force Space

**Figure 10:**
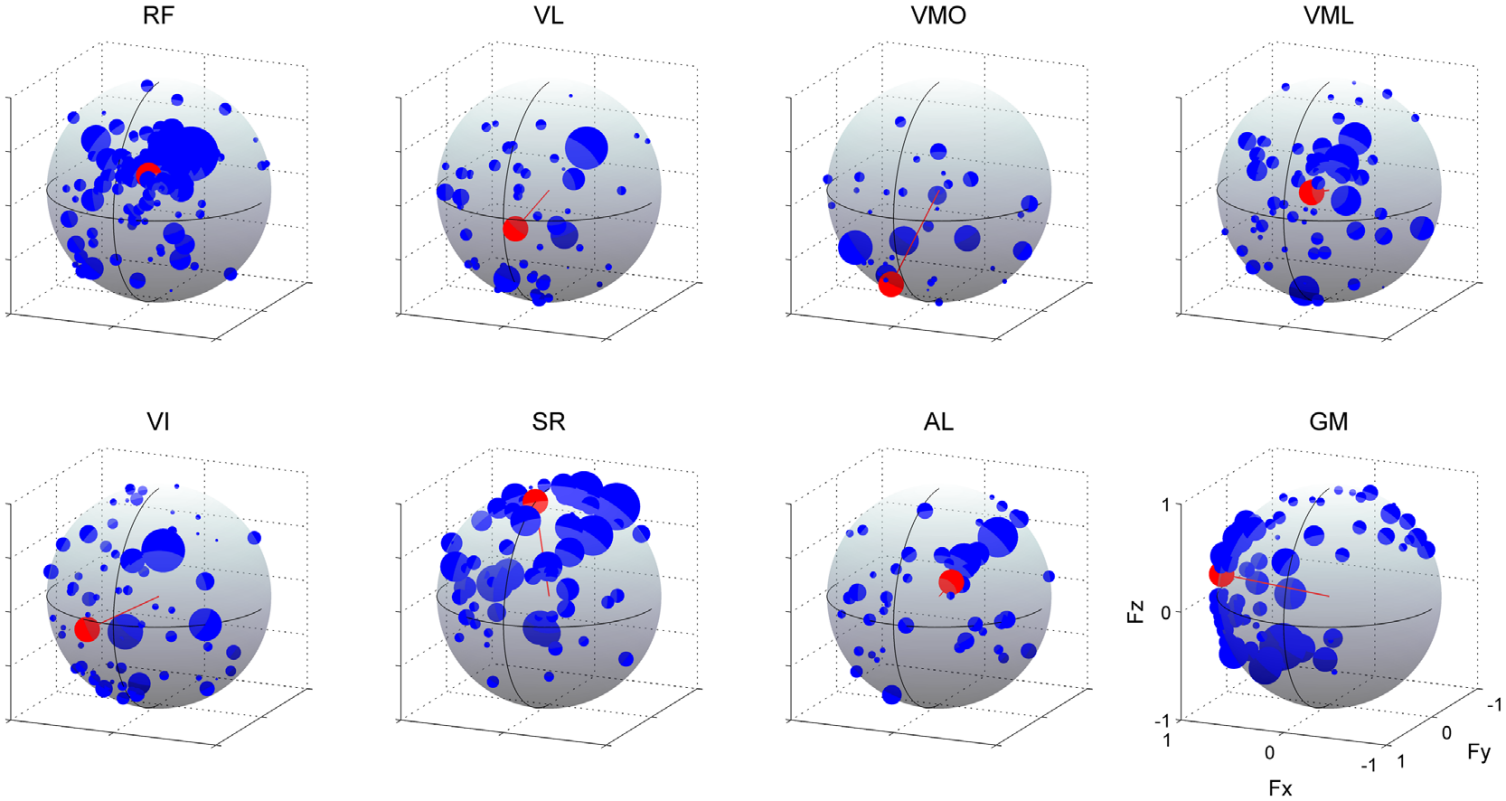
**Action directions of muscles**. The ADs (red) and force vectors (blue) across each muscle. The length of each vector was uniformed with their unit vectors, and the correlation coefficient was described as the radius of each plot distributed on the surface of unit sphere. Data shown are for all subjects and selected trials (see detail in Section “Materials and Methods”). The positive values of three axes are corresponding to hip abduction (*F*_x_), knee extension (*F*_y_), and hip flexion (*F*_z_) movement directions, respectively.

As illustrated in Figure 10, we estimated the AD of each muscle (red) to validate the hypothesis that neural-based muscle synergy would synchronously control organized muscles. We verified the significance in the distribution of the force vectors across each muscle (*p* < 0.05; see detail in Section “Materials and Methods”). These ADs were approximately corresponding to the range of the muscle activation direction. However, they represented the characteristics of each muscle more distinctly. Although VL, VMO, VML, and VI, mono-articular knee extensors, are generally assumed as functionally similar muscles, the ADs were different among them. The AD of VL denoted the force direction generated by both knee extension and small hip flexion torques [(−0.113, 0.990, −0.086); (*F*_x_, *F*_y_, *F*_z_)]. This contribution to the off-axis hip joint torque would be due to the synchronous activation with RF, which generates knee extension and hip flexion torques, in the synergy *W*_5_. The similar result was observed in VI (0.274, 0.958, −0.088). On the other hand, the AD of VMO was around the direction produced only by the knee extension torque (0.166, 0.680, −0.714) because VMO was contained only in the synergy *W*_1_, which AD (0.215, 0.680, −0.701) was similar to the AD of VMO, and not associated with the bi-articular muscle, RF. The AD of VML was in the force direction produced by both knee extension and hip flexion torques (−0.245, 0.936, 0.252) and was strongly similar to the AD of RF (−0.288, 0.871, 0.398), both of which was included in the synergy *W*_2_. Interestingly, the ADs of the knee extensors also directed either medial or lateral side, indicating the synchronous activations of the hip adductor, AL, or hip abductor, GM, through the synergy *W*_5_ and *W*_4_, respectively. The ADs of the SR, AL, and GM were also affected by the synchronization with the different muscles in the same muscle synergies [SR (0.002, 0.298, 0.955), AL (−0.470, 0.790, 0.393), and GM (0.977, 0.164, 0.135)]. These results suggest that the AD of a muscle reflect the anatomical function of the muscle and different muscles, which are synchronously activated through the muscle synergies.

**Figure 11:**
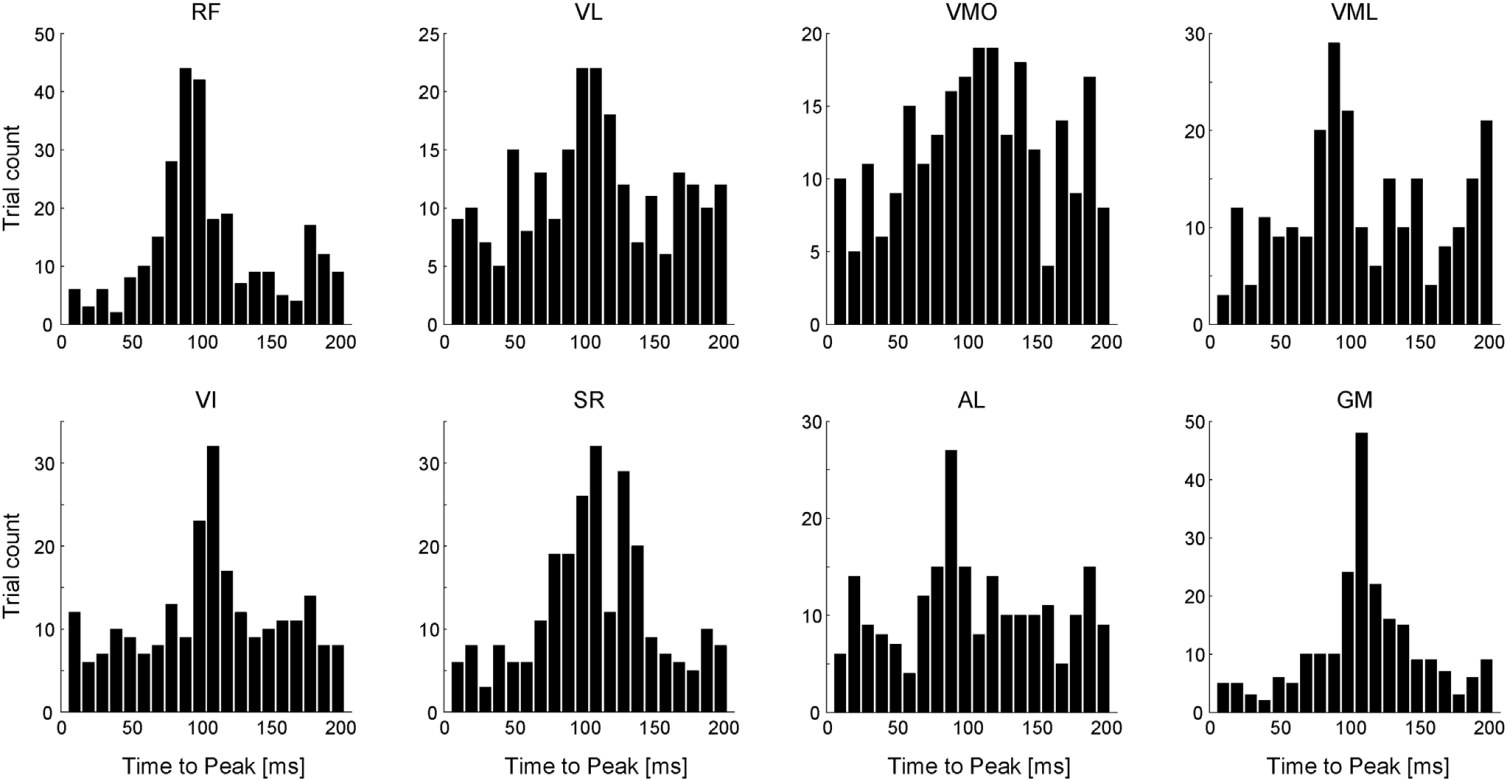
**Electromechanical delay (EMD)**. Histograms of time-to-peak value across each muscle referred to as the time lag between the onset of EMG and mechanical force responses. Data shown are for all trials and all subjects.

To verify the physiological validity of the ADs, we calculated the EMD of each muscle. Figure 11 shows the EMD histograms of each muscle for all analyzed trials at a time lag of 0 to 200 ms. Each muscle had the peak time bin around a time lag of 100 ms [102.5, 103.7, 104.8, 107.4, 103.9, 104.7, 101.9, and 108.5 (ms); RF, VL, VMO, VML, VI, SR, AL, and GM, respectively). The average time-to-peak values were similar to the values of the previous study (Vos et al., 1990), indicating that the estimated ADs in this study would be physiologically valid.

## Discussion

The primary goal of this study was to quantify the mechanical contribution of muscle synergies in the task space. To this end, we estimated the AD of lower limb muscle synergies during isometric force-maintaining tasks on three-dimensional force space. The five or six muscle synergies were identified across each subject. The ADs of these muscle synergies approximately denoted the direction based on the anatomical function of the weighted muscles in the task space. Furthermore, the AD of each muscle reflected each anatomical function and a synchronous contribution with different muscles, which belonged to the same muscle synergies.

### Action Direction of Muscle Synergies

Many researchers have long studied low-dimensional organization of the spinal motor system and resulting movements. Low dimensionality in the task space, which was induced by the stimulation of spinal interneuron, was first observed in frog and rat as force field (Giszter et al., 1993; Saltiel et al., 1998). Modular organization of muscle activations (so-called muscle synergy) was then statistically estimated (Tresch et al., 1999), which would produce the low dimensionality in the task space. A few studies showed the relationship of the low dimensionality between task level and motor level. Novel method was conducted to estimate synergy-to-force-mapping, which represented the linear relationship between the activation of muscle synergies and endpoint force in the isometric condition (Berger et al., 2013; Berger and d’Avella, 2014), whereas synergy-to-force-mapping vector did not contain the contribution of unmesurable muscles and did not completely explain the net contribution of muscle synergies in the task space. Different approach calculated functional muscle synergies from the data matrix, which contained muscle activation matrix and corresponding endpoint force matrix, by reducing their dimension together using NMF algorithm (Torres-Oviedo et al., 2006; Chvatal et al., 2011). The counter evidence of the muscle synergy hypothesis, however, showed that the estimated low-dimensionality in muscle activation was due to the biomechanics of the limb, which constrains musculotendon length changes (Kutch and Valero-Cuevas, 2012). Thus, the constraints of limb geometry relevant to task space can lead to the low dimensionality in the measured EMG activity. Therefore, the identification of neural-based muscle synergies will require examining not only the spatial contributions of muscle synergies in the task space but also temporal contributions, which include both movement-based and neural-based fluctuations (Hagio and Kouzaki, 2015). For this reason, the previous studies only evaluating the spatial contribution of muscle synergies could not provide the direct evidence whether the estimated contribution in the task space was arose from neural-based muscle synergies (Torres-Oviedo et al., 2006; Chvatal et al., 2011; Berger and d’Avella, 2014). In this study, we could directly estimated the net contribution of muscle synergies in the task space while considering temporal correlation between the activation of muscle synergies and endpoint force in the basis of the physiological criteria, i.e., time delay from the onset of the muscle synergy activation to the resulting force (indeed, this delay was between the onset of the muscle activation constructing muscle synergies and the force) (Figure 7). Our method in the basis of the previous technique (Kutch et al., 2010) made it possible to regard muscle synergies as neural basis and to directly quantify the spatiotemporal contribution of muscle synergies to the endpoint force.

Variability was observed across each force vector constructing AD of muscle synergy (Figure 7C; blue dots). The possible reason of this variability was due to synchronous activation with the other muscle synergies. In the methodology, the previous study showed that synchronization of motor units having different pulling directions distorts the estimate of the pulling direction by STA (Kutch et al., 2007). On the other hand, in the physiological aspect, the merging of muscle synergies was observed depending on the force-generating capability of muscles, which might result from the simultaneous recruitment of a few different muscle synergies (Hagio and Kouzaki, 2014). In the basis of the fact, the AD would be estimated by the correlation between a target muscle synergy and endpoint force, which was generated by the combination of the target muscle synergy and the synchronously activated muscle synergies. The different possible reason of this variability attributed the mechanical property of motor units. If the neural basis of muscle synergies exists as spinal interneuron (Hart and Giszter, 2010; Overduin et al., 2014), these interneurons control individual motor units having a broad range of the pulling direction (Thomas et al., 1986, 1990). This fact suggests that the mechanical contribution of muscle synergies was varied depending on the recruited motor units, which were activated according to a neural property, such as the Henneman’s size principle: if the neural input from spinal interneuron to motor units was increased, motor units are recruited in turn from the smallest to the largest. This variability could make it possible for flexible force generations in a broad range of the task space by the combination of a small number of muscle synergies (Roh et al., 2012; Hagio and Kouzaki, 2014). These results also suggest that the ADs of muscle synergy defined in this study represent the average of variability of pulling direction of muscle synergies arose from a range of the pulling direction of motor units. Therefore, not simple combinations of ADs of different muscle synergies but flexible modulation within a muscle synergy might determine the produced endpoint forces.

### Action Direction of Muscles

The second effort in this study was to provide evidence that muscle synergies were of neural origin. To this end, we hypothesized that the AD of each muscle reflects the mechanical contribution of different muscles, which belong to the same muscle synergy, based on the consideration that if muscles are synchronously activated by the muscle synergies, cross-correlation analysis leads to the correlation between the activation of the target muscle and the endpoint force generated by the combination of these muscles. Indeed, the ADs of knee extensors (VL, VMO, VML, and VI) were different from each other depending on the muscle synergies, which these muscles belong to, despite their similar anatomical function (Figure 10). The results indicated that the muscles spanning different joints, such as bi-articular, RF, AL, and GM, affected the ADs of these muscles. The previous studies conducted the novel method focusing on the synchronous recruitment of each muscle through muscle synergies and showed the low-dimensional structure in the EMG activity (Krouchev et al., 2006; Drew et al., 2008; Krouchev and Drew, 2013). The extracted clusters, however, were relatively more than the estimated muscle synergies using decomposing technique, such as NMF (Krouchev et al., 2006). The method and idea in the present study could demonstrate the synchronous recruitment of muscles due to muscle synergies extracted by NMF. Moreover, because the method focused on both high and low force fluctuations during constant force generation, the correlation might reflect not the co-contraction of muscles but the synchronization of motor units constructing the measured EMG signals, which have the innervation from the same muscle synergy. This temporal property of muscle synergies provides the provided evidence that muscle synergies are of neural origin.

The estimation of muscle ADs also provided the EMD of each muscle, i.e., the time lag between the EMG and mechanical force response, which peak of the distribution was different across each muscle. Muscle synergies were composed of any muscles, which EMDs were variable. This difference among EMDs apparently confounds a motor control because the mechanical responses induced by descending neural input to muscle synergy may be out of alignment among muscles. Each muscle synergy, however, had roughly constant peak time lags between the activation of muscle synergy and force responses (Figure 7D). This result implies that the motor units having the similar EMDs in a muscle or in more different muscles compose a muscle synergy. Therefore, muscle synergies are constructed considering the complexity in the misalignment of EMDs and achieve the accurate force generations. In the different scheme, such temporal lag between muscles within the same synergy was previously observed as “time-varying synergies,” which has a fixed temporal profile (d’Avella et al., 2003), suggesting that this delay consider the difference of EMDs among muscles.

Furthermore, the difference of the muscle ADs among functionally similar muscles, especially between VML and the other vasti muscles (VL, VMO, and VI) (Figure 10), might reflect not only the modularity due to muscle synergies but also the inherent relationship between muscles and force responses based on the intrinsic characteristics in the musculoskeletal system. It is known that the EMG activities between VML and VL were different because of the discordancy of physiological parameters, such as physiological cross-sectional area (PCSA) and pennation angle of muscle fiber (Akima et al., 2000, 2001; Ward et al., 2009; Watanabe and Akima, 2011) or contribution to torque (Zhang et al., 2003). Moreover, this result could reflect the divergence of relationship between these muscles and the bi-articular muscle, RF, suggesting the stronger association between RF and VML than the other knee extensors. On the other hand, the ADs of VL and VI were mainly distributed in the same area. As illustrated in Figure 7A, the synergy *W*_1_ mainly consisted of the mono-articular knee extensors, in which the weightings of VL and VI were similar to each other. It is reported that they are fused at posterolateral side (Willan et al., 1990) or have relatively equivalent physiological parameters, such as PCSA (Akima et al., 2000, 2001). Hence, this result reflects the morphological and physiological similarity between VL and VI. Additionally, it is generally accepted that the principal function of VMO is to control tracking of the patella by overcoming the lateral forces imposed by the other vasti muscles. This fact led us to the speculation that VMO does not have the specific AD. This fact was also ensured by the incoherent time-to-peak value of VMO (Figure 11). The calculated AD, which directed the net knee extension directions, might reflect the association between VMO and VL, which applies lateral-directed forces to the patella.

Furthermore, the AD of VI and VMO also directed to the lateral, whereas RF, VL, and VML contributed to medial force. The similar result was previously reported and suggested that balanced off-axis torques and forces are necessary for appropriate three-dimensional patellar tracking and tibiofemoral movement, and different quadriceps components need to be coordinated to generate appropriate off-axis and extension torque around knee joint (Zhang et al., 2003). Therefore, the ADs in the three-dimensional force space reflected such complicated relationships of quadriceps muscles.

### Existence of Hard-Wired Muscle Synergies

The primary problem in the muscle synergy hypothesis is whether a muscle synergy is a hard-wired neural system. Many researchers addressed the problem in some empirical studies. Hart and Giszter (2010) showed that activation of spinal interneurons in frogs was related to statistically calculated muscle synergies rather than individual muscles, indicating the neural-based structure of a muscle synergy as a spinal interneuron (Hart and Giszter, 2010). In addition, the connectivity between motor cortical neurons and muscle synergies was demonstrated by testing the similarity between statistically extracted muscle synergies evoked by intracortical microstimulation and hand movements in rhesus macaques (Overduin et al., 2012, 2014). In humans, a virtual surgery technique that rearranged muscle architecture demonstrated a hard-wired modularity in a neural circuit by testing the prediction that modularity due to muscle synergies interfered the adaptation to perturbations that are incompatible with the muscle synergies (Berger and d’Avella, 2014). However, a low-dimensional structure as statistically calculated muscle synergies might be a secondary product due to some decomposing techniques, such as NMF or independent component analysis (Bell and Sejnowski, 1995; Hart and Giszter, 2004), in task or biomechanical constraints (Kutch and Valero-Cuevas, 2012). Accordingly, to reveal the problem whether a muscle synergy is of neural origin, it is important to focus on not only the spatial but also the temporal structure of muscle synergies, which comprehended information of both a mechanical and a neural property. In this study, we demonstrated the mechanical contribution of the muscles, which activation was synchronized with the different muscles, due to a modular control of muscle synergies (Figure 10). Furthermore, the procedure, which considered temporal correlation between activation traces of muscle synergies and endpoint force fluctuations containing various information of neural property (Hagio and Kouzaki, 2015), enabled to estimate the ADs of muscle synergies (Figure 7). These results suggest that low dimensionality in muscle space was due to not simply a biomechanical constraint but a neural constraint as a hard-wired muscle synergy.

In summary, we could quantify the mechanical contribution of lower limb muscle synergies during isometric force-generating tasks in three-dimensional force space around a right ankle as considering spatiotemporal correlation between activation of muscle synergies and endpoint force. Furthermore, the ADs of knee extensors were different despite functionally similar muscles, which depended on the other muscles weighted by the same muscle synergies, suggesting that muscles were synchronously activated through a hard-wired constraint as a muscle synergy. These results provide strong evidence that neural-based muscle synergies spatiotemporally contribute to the low-dimensional force generation in a task space.

## Author Contributions

Conception and design of the experiments: SH and MK. Collection, analysis, and interpretation of data: SH. Drafting the article or revising it critically for important intellectual content: SH and MK. Final approval of the version to be published: SH and MK.

## Conflict of Interest Statement

The authors declare that the research was conducted in the absence of any commercial or financial relationships that could be construed as a potential conflict of interest.
